# Nagaoka ferromagnetism in an array of phosphorene quantum dots

**DOI:** 10.1038/s41598-023-45860-3

**Published:** 2023-11-01

**Authors:** Tanmay Thakur, Bartłomiej Szafran

**Affiliations:** Faculty of Physics and Applied Computer Science, AGH University of Krakow, al. Mickiewicza 30, 30-059 Kraków, Poland

**Keywords:** Quantum dots, Two-dimensional materials

## Abstract

We consider an array of four quantum dots defined in phosphorene containing three excess electrons, i.e., in the conditions of near half filling when itinerant Nagaoka ferromagnetism is expected to appear in a square array with isotropic interdot hopping. The interdot hopping in the array arranged in a square inherits the anisotropy from the form of the phosphorene conduction band. We apply the configuration interaction method for discussion of the appearance and stability of the spin-polarized ground state and discuss the compensation of the effective mass anisotropy by the geometry of the quantum dot array. Our study shows strong stability of Nagaoka ferromagnetism for optimized geometry of the array, with the Nagaoka gap as large as ∼ 230 µeV. A phase diagram for the ground-state spin ordering versus the geometric parameters of the array is presented. We study the suppression of the ferromagnetism in a transition of the $2\times 2$ array to a quasi-1D chain and indicate that the shift of one of the quantum dots away from the array center is enough to transform the system to a quantum dot chain. A shift in the zigzag crystal direction induces the low-spin ground state more effectively than a shift along the armchair direction. We also discuss the robustness of the spin ordering against detuning one of the dots. The ferromagnetic ground-state survives as long as the detuning is not large enough to trap one of the electrons within a single quantum dot (for positive detuning) or remove one of the quantum dots of the accessible energy range (for negative detuning).

## Introduction

The Hubbard model has been a catalyst for the development of novel and fascinating physics since it first appeared. Although originally meant to provide an understanding of itinerant ferromagnetism in transition metals^1^, the variants of this model explain complex phenomena like Mott transition^2^, superconductivity^3,4^ , ferrimagnetism^5^ , antiferromagentism^6^ , etc. The model has not only been used to theoretically investigate strongly correlated systems, but also been physically implemented in the form of quantum simulations utilizing quantum dots in semiconductors^7,8^. The quantum dot systems are a playground for verification of the results of the Hubbard model experimentally. One of the results is Nagaoka ferromagnetism predicted for the ground state of single-band systems nearly half filled by strongly interacting electrons^9,10^. Complete spin polarization of the system is favorable to reduce kinetic energy due to interference of the hole hopping paths in the array^11^. The Nagaoka ferromagnetism was experimentally demonstrated using a square quantum dots system in GaAs^11^ validating the theoretical results^12,13^ for a 2D array. Small systems such as quantum dot plaquettes as in Ref.^12^ are feasible systems to test and investigate the Nagaoka theorem in depth. The experimental realization of such systems still requires that the energy gap be at least a few $\mu$eV and its tolerance to disorder and imperfections.

Recently, black phosphorene^14^ a monolayer form of black phosphorus has been on the rise and is extensively studied for its strongly correlated phenomena^15–18^. The anisotropic characteristics and tunability of its optical properties by the number of layers make black phosphorene an attractive candidate for applications such as optoelectronics^19–22^. The material is particularly interesting in the context of electron hopping within the array due to the highly anisotropic carrier effective masses^23–25^. Additionally, the effective mass of the electrons in phosphorene is much higher than that of GaAs. The large effective electron mass reduces the contribution of kinetic energy, and then the physics is largely affected by electron–electron interactions, which makes quantum dots in phosphorene an appealing choice for studies of the interaction-driven Nagaoka ferromagnetism.

The Hubbard model for GaAs in Ref.^11^—an isotropic effective mass material—employs isotropic hopping between the sites (i.e. the quantum dots) that is not suitable for phosphorene. The anisotropy in the effective masses in *x* and *y* directions will lead to the anisotropy of the hopping integrals. Although, investigation of a rectangular geometry with anisotropic hopping has also been reported^26^. Nevertheless, accurate results based on the anisotropic effective mass model are missing. The purpose of this paper is to fill this gap. For phosphorene, the anisotropy of the effective mass calls for an anisotropic geometry of the array, which in turn induces anisotropy of the interdot interaction energy. We show that the Nagaoka gap in the quantum dot plaquette in phosphorene is greater than just a few $\mu$ eV and therefore verification should be within the experimental reach. We illustrate the robustness of this state against different conditions, revealing the underlying physics, using the configuration interaction method with the effective mass Hamiltonian^24^ for phosphorene.

The paper is organized as follows: First, we establish the theory and describe the computational approach in detail. In Section “Nagaoka spin polarization” we optimize the parameters and the geometry to get the largest Nagaoka gap. We discuss the ground-state phase diagram with respect to the array parameters. Next, in “Shift from the rectangular geometry” section we discuss the physics of the transition of the rectangular structure of dots to a pseudo-chain structure. In the last subsection of the results, we detune one of the potential wells to simulate the effect of system imperfection and test the Nagaoka phase against it. Lastly, the conclusions and summary of the work are given in Section “Summary and conclusion”.

## Theory

In this section “Single electron” we describe the applied continuum Hamiltonian for phosphorene. The system parameters and the potential applied for the quantum dots are discussed in Section “Model potential” and finally, the calculation methods and techniques are elaborated in Section “Three-electron Hamiltonian and electron density”.

### Single electron

**Figure 1:**
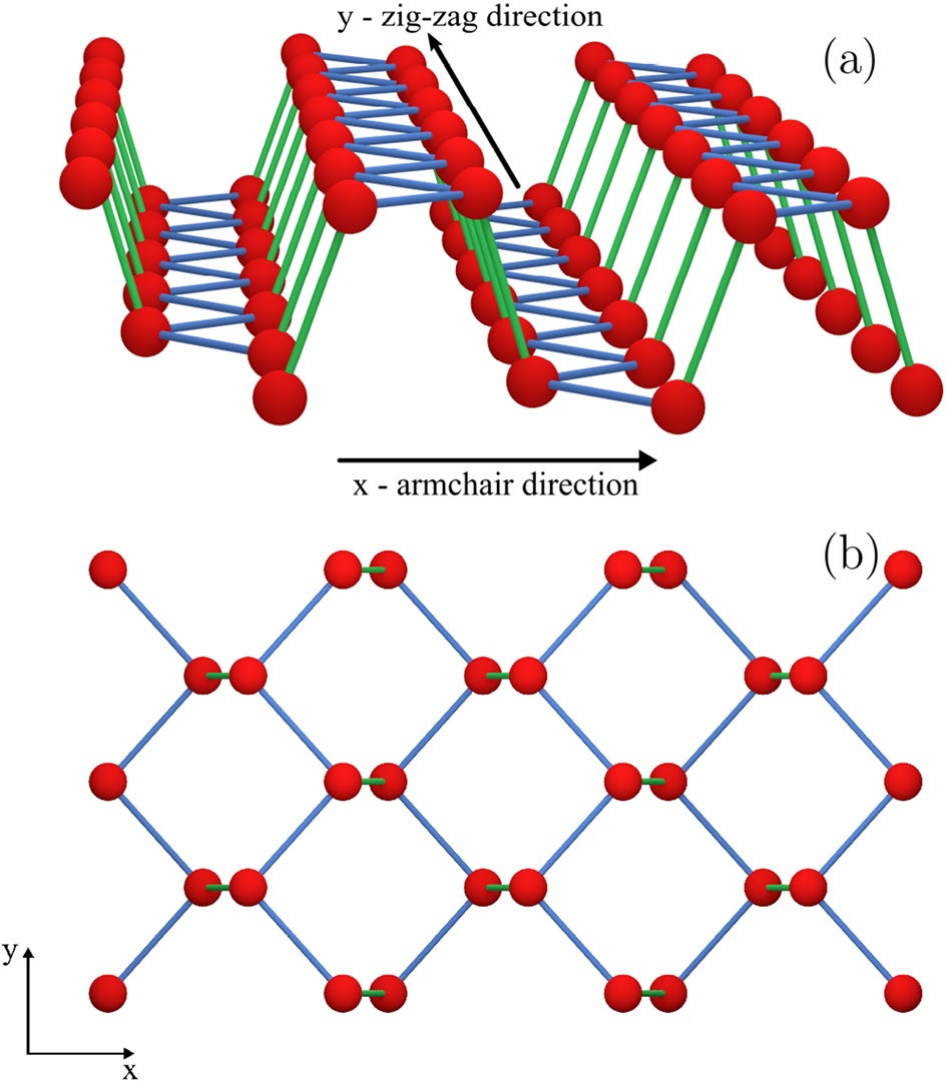
(**a**) Crystal structure of monolayer phosphorene indicating the zigzag direction (y-axis) and the armchair direction (x-axis). (**b**) Top view of the phosphorene crystal.

An electron in the phosphorene monolayer can be described with the single-band continuum Hamiltonian^24,27^,

1 $$ \begin{aligned}   H_{0}  =  & \left( { - i\hbar \frac{\partial }{{\partial x}} + eA_{x} } \right)^{2} /2m_{x}  + \left( { - i\hbar \frac{\partial }{{\partial y}} + eA_{y} } \right)^{2} /2m_{y}  \\     &  + g{\mkern 1mu} \mu _{B} B{\mkern 1mu} \sigma _{z} /2 + V(x,y), \\  \end{aligned}  $$

with effective masses $m_x=0.17037m_0$ along the armchair direction (see Fig. 1) and $m_y=0.85327m_0$ along the zigzag direction, derived using a tight-binding Hamiltonian in Ref.^24^ and *V*(*x*, *y*) is the confinement potential. The effective masses were obtained by fitting the energy spectra of the single-band continuum Hamiltonian to the tight-binding model for the harmonic oscillator^24^ and the ring-like confinement potential^27^. The magnetic field is applied perpendicular to the plane of 2D phosphorene crystal for non-zero Zeeman term and is introduced with symmetric gauge $\textbf{A}=(A_x,A_y,A_z)=(-yB/2,xB/2,0)$. In Eq. (1), we use the Landé g-factor, $g=g_0=2$. The value is taken from Ref.^28^ where $k\cdot p$ theory was used to extract the Landé g-factors. The purpose of including the Zeeman term in the Hamiltonian is to remove the degeneracy of spin eigenstates. A range of *g* values were reported^29–32^. The specific value of *g* changes the magnitude of the magnetic field required to lift the degeneracy. We introduce the external magnetic field for a residual lifting of the spin degeneracy which is useful for the characterization of the spectrum. In the discussion of Nagaoka ferromagnetism, for convenience, we set a residual magnetic field $B=1$ mT to lift the degeneracies with respect to the *z* component of the spin $\sigma _z$. The Nagaoka gap measured experimentally for the GaAs system was of the order of a few $\mu$eVs^11^. We expect our system to have a much larger gap for the strongest ferromagnetic state. The magnetic field produces splitting between states of the *z* component of the total spin $\sigma _z=-3/2$ and $\sigma _z=-1/2$ of about 0.22 $\mu$eV. As we will see in further results, this energy will be small compared to the largest Nagaoka gap, and will affect the ground state only when the Nagaoka gap is of the same order (near the phase transition) and not the results near the point of largest gap. The energy gap is calculated as $\Delta E =E_{3/2}-E_{1/2}$ where $E_{3/2}$ is the energy of the spin quantum number $S=3/2$ state and $E_{1/2}$, the energy of the spin $S=1/2$ state.

The Hamiltonian in Eq. (1) is solved using the finite difference method with gauge-invariant discretization^33^. We use a square mesh with spacing $\Delta x$ in both directions so that the action of Hamiltonian on the wave-function $\Psi _{\alpha ,\beta }=\Psi (x_\alpha , x_\beta )=\Psi (\alpha \Delta x, \beta \Delta x)$ is given by

2 $$ \begin{aligned}   H_{0} \Psi _{{\alpha ,\beta }}  \equiv  & \frac{{\hbar ^{2} }}{{2m_{x} \Delta x^{2} }}\left( {2\Psi _{{\alpha ,\beta }}  - C_{y} \Psi _{{\alpha ,\beta  - 1}}  - C_{y}^{*} \psi _{{\alpha ,\beta  + 1}} } \right) \\     &  + \frac{{\hbar ^{2} }}{{2m_{y} \Delta x^{2} }}\left( {2\Psi _{{\alpha ,\beta }}  - C_{x} \Psi _{{\alpha  - 1,\beta }}  - C_{x}^{*} \psi _{{\alpha  + 1,\beta }} } \right) \\     &  + V_{{\alpha ,\beta }} {\mkern 1mu} \Psi _{{\alpha ,\beta }}  + \frac{{g\alpha _{B} B}}{2}\sigma _{z} \Psi _{{\alpha ,\beta }} , \\  \end{aligned}  $$

with Peierls phases^33^ $C_x=\exp (-i\frac{e}{\hbar }\Delta x A_x)$ and $C_y=\exp (-i\frac{e}{\hbar }\Delta x A_y)$ to account for orbital effects of the magnetic field. The Hamiltonian in Eq. (2) is diagonalized in a finite computational box of size 50 nm $\times \, 50$ nm and the spacing $\Delta x$ of 0.4 nm. The numerical diagonalization is carried out using the Lanczos algorithm as in Ref.^24^.

### Model potential

Inside the computational square box of side length 50 nm, the model quantum dots are arranged in a rectangle with distance between the centers of dots $2\mu _x$ in the armchair direction (*x*) and $2\mu _y$ in the zigzag crystal direction (*y*). The spacings influence both the interdot tunneling rates (hopping energy) and the interdot electron–electron interaction. The applied model confinement potential is

3 $$\begin{aligned} V(x,y)= & {} -V_d \, \sum _{i=1}^2 \, \sum _{j=1}^2 \exp \left( \frac{ -(x+(-1)^i \mu _x)^2}{s^2}\right) \\     &  \cdot exp \left( {\frac{{ - (y + ( - 1)^{j} \mu _{y} )^{2} }}{{s^{2} }}} \right){\mkern 1mu}  \\  \end{aligned}$$

such that the centers of the dots are located at $(\pm \mu _x,\pm \mu _y)$. Each dot is determined by a Gaussian potential with the size given by the parameter *s* and the depth of the potential by $V_d$. Throughout the work, the size of the dots is taken to be $s=7$ nm. The chosen soft Gaussian potential is a good approximation for the electrostatic quantum dots^34^ and the construction of four such electrostatic quantum dots setup should be within the experimental reach with the help of a pair of flat gate electrodes with circular intrusion near the center of the dots^35–37^.

### Three-electron Hamiltonian and electron density

The energy spectrum for three interacting electrons in our system is calculated in a continuum approach using the following Hamiltonian

4 $$\begin{aligned} H_{3e}=\sum _{i=1}^3 H_0(i)+\sum _{j>i}^3 \frac{e^2}{4\pi \epsilon _0\epsilon \, r_{ij}} \,, \end{aligned}$$

where $H_0$ is a single electron Hamiltonian in Eq. (1) and the dielectric constant has the value $\epsilon =9$ for all calculations in the work. The present study calls for an exact treatment of the electron–electron correlation due to the strong electron–electron interaction in phosphorene^29^. We employ the configuration interaction approach^38,39^. In this method, the Hamiltonian $H_{3e}$ is diagonalized in the basis of three-electron Slater determinants constructed with the lowest 52 eigenfunctions of the single-electron Hamiltonian $H_0$. The Slater determinants are built by filling the three electrons in the single-electron states in all available combinations. The three-electron ground state and excited states are then written as the linear combination of these configurations of electrons. The number of single-electron eigenfunctions spanning the basis of the Slater determinants was chosen on the basis of convergence of the three-electron energy eigenvalues. For 52 single-electron eigenfunctions, the basis of three-electron Slater determinants contains ${52 \atopwithdelims ()3}$ = 22100 elements. The Hamiltonian matrix is then numerically diagonalized to obtain three-electron eigenfunctions and corresponding energies.

We investigate the electron distribution in the dots by extracting the electron density from the three electron wave-functions $\Psi$,

5 $$\begin{aligned} \rho (\textbf{r}) = \langle \Psi | \, \sum _{i=1}^3 \delta (\textbf{r}-\textbf{r}_i) \, | \Psi \rangle \end{aligned}$$

where $\Psi$ is the three-electron ground-state wave function.

## Results

We discuss our results in the following order: First, we discuss the parameters of the system for the energy spectrum and the electron density, including the spin ground-state configuration. Next, we calculate a complete phase diagram for the nonferromagnetic and Nagaoka ferromagnetic phases. This gives us insight on the configuration for which the Nagaoka ferromagnetism is the most robust. We then discuss a similar phase diagram but with a shallower potential. We investigate the case where we gradually change the configuration of dots to form a pseudo-1D chain of dots to understand the competition between the ferromagnetic and nonferromagnetic states and the effect of anisotropy on this competition. Lastly, we observe the effect on the Nagaoka phase of a changed potential of one of the quantum dots.

### Nagaoka spin polarization

**Figure 2:**
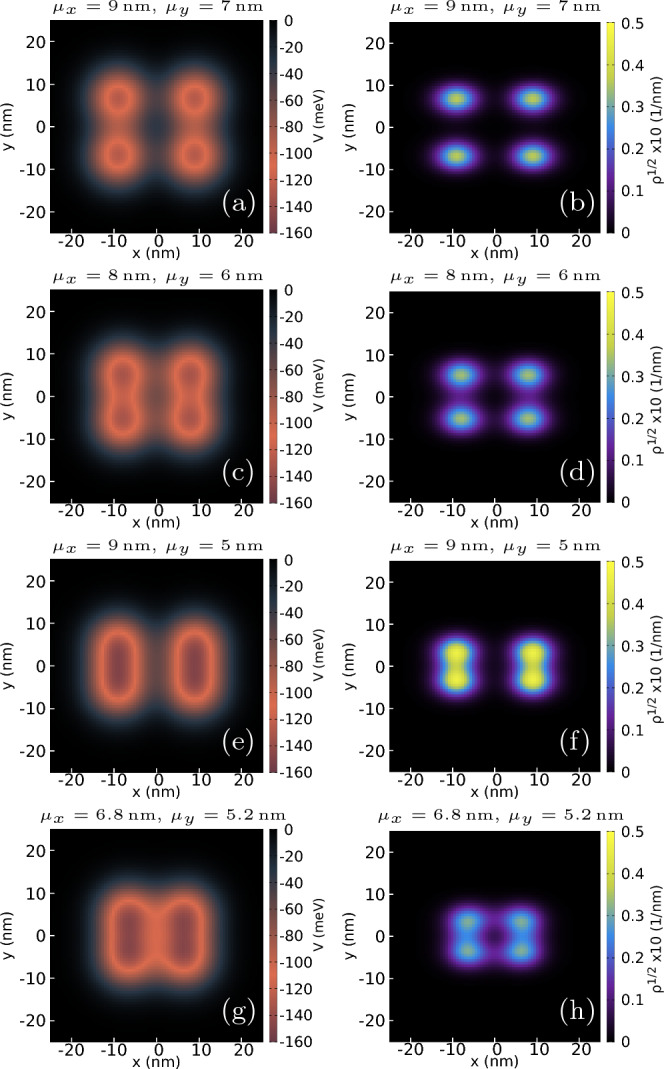
The potential (left column) and the square root of electron density (right column) with parameters, $\mu _x=9$ nm, $\mu _y=7$ nm (**a**,** b**); $\mu _x=8$ nm, $\mu _y=6$ nm (**c**,** d**); $\mu _x=9$ nm, $\mu _y=5$ nm (**e**,** f**); $\mu _x=6.8$ nm, $\mu _y=5.2$ nm (g, h). For all the cases, the magnetic field is 1 mT and the potential depth $V_d=125$ meV. The color scale for each plot is to the right of each plot. Subfigures (**g**,** h**) indicate the potential and square root of the density for the configuration of dots with the largest Nagaoka gap.

The anisotropy of the effective mass causes the *x* and *y* axes of the system to be inequivalent. Therefore, we vary both parameters, $\mu _x$ and $\mu _y$ to search for the high-spin configuration in the ground state. The potential and square root of the density of the ground state for different combinations of the parameters are plotted in Fig. 2. The potential depth considered for the following calculations is $V_d=125$ meV. The densities are calculated using Eq. (5) for the ground state. For spacings $\mu _x = 9$ nm, $\mu _y = 7$ nm (Fig. 2a) the electrons are located near the center of four dots, the charge densities of the array are distinctly separated with little or no density overlap (Fig. 2b). Note that even though the dots are far away, the square root density in the center of dots is not circular, but is more spread in the *x* direction (Fig. 2a), which is a consequence of the mass anisotropy. For the rest of the plots with $\mu _y\le 6$ nm the potential [see Fig. 2c,e,g] resembles two dumbbell-like shapes due to the electron tunneling in the zigzag direction, for which the assumed distance between the dots is smaller than in the armchair direction. The trace of electron tunneling between the dots can be observed in Fig. 2d for $\mu _x = 8$ nm, $\mu _y = 6$ nm. As we move the dots closer together in the *y* direction, we can observe in Fig. 2f that the square root densities of the top-bottom pair of dots now overlap considerably. Finally, for dots arranged in a rectangle with $\mu _x = 6.8$ nm, $\mu _y = 5.2$ nm, the square root electron density is highest along the edges of the rectangle.

**Figure 3:**
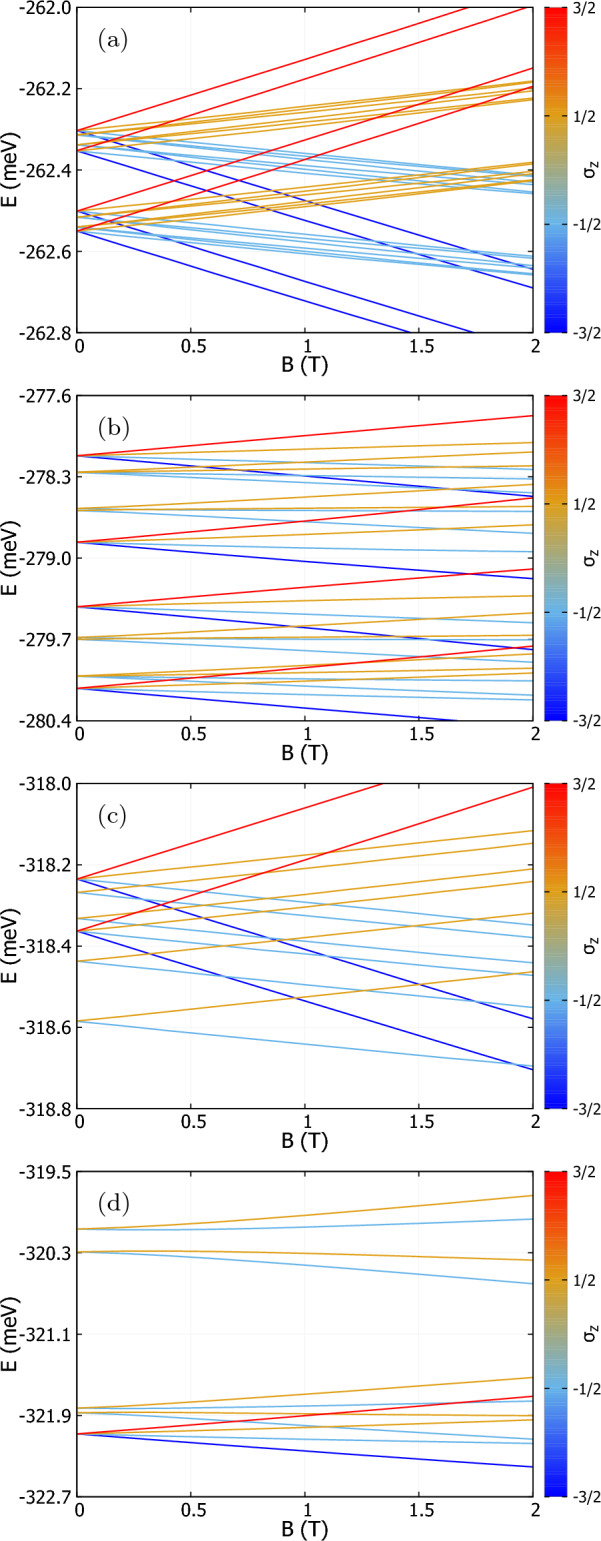
The energy spectrum as a function of magnetic field *B* for parameter sets of $\mu _x=9$ nm (**a**), $\mu _y=7$ nm, (**b**) $\mu _x=8$ nm, $\mu _y=6$ nm, (**c**) $\mu _x=9$ nm, $\mu _y=5$ nm, (**d**) $\mu _x=6.8$ nm, $\mu _y=5.2$ nm. Potential depth is set $V_d=125$ meV for all the chosen sets in (**a**–**d**). The colour of each line denotes the spin eigenvalue of the energy state, and the scale is shown to right of each plot. The spectra show the shifting of ground states and the changing Nagaoka gap as the configurations of the dots change. Subfigure (**d**) shows the spectrum for the configuration with the largest Nagaoka gap.

The energy spectra for the same parameters are plotted in Fig. 3. For $\mu _x = 9$ nm and $\mu _y = 7$ nm, the ground state at $B=0$ is a four-fold degenerate state with spin eigenvalue $S=3/2$ but the spin doublet 1/2 is above with the energy difference of $40\,\mu$eV (Nagaoka gap $\Delta E=-40\,\mu$eV). The low-energy spectra are illustrated in Fig. 3a. Although the tunneling coupling between the dots is weak, it is already large enough to promote the spin-polarized quadruplet with the total spin eigenvalue of $S=3/2$ and the *z* component eigenvalues $\sigma _z=-3/2,-1/2,1/2,3/2$ in the ground state. An asymptotic case of large interdot distances corresponds to tunneling and interaction being negligible so that the quadruplet becomes degenerate with the spin $S=1/2$ doublet.

Reducing the spacing between the dots to $\mu _x = 8$ nm and $\mu _y = 6$ nm, the energy gap between the lowest spin $S=1/2$ and spin $S=3/2$ states becomes $100\,\mu$eV with the high-spin ground state [see Fig. 3b].

The energy spectrum for $\mu _x = 9$ nm and $\mu _y = 5$ nm is plotted in Fig. 3c. In this case, the electrons interact weakly in the *x*-direction and strongly in the *y*-direction. We have effectively two extended quantum dots with an electron in one of the other two dots. Since the ground state of the two electrons for negligible magnetic field is a singlet state^40^, the top-bottom pair of dots will contain two electrons of opposite spins, resulting in the ground state being a spin $S=1/2$ state due to the spin of the solitary electron. The ground state becomes spin-polarized by the Zeeman interaction only at a high magnetic field of about 1.9 T.

The spin singlet state is removed from the ground state when the tunneling in the *x* direction is enhanced for a reduced distance in *x* direction to $\mu _x=6.8$ nm with $\mu _y=5.2$ nm we obtain the ground state, which is again spin polarized with a large energy gap of $\Delta E=-230\,\mu$eV in Fig. 3d. This is the maximal gap that we obtain for the applied single-dot potential depth.

**Figure 4:**
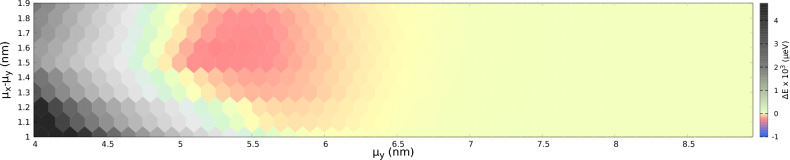
The energy gap, $\Delta E$ as a function of the parameters $\mu _y$ and $(\mu _x-\mu _y)$ with potential depth, $V_d=125$ meV. The energy gap is calculated for every point at the center of the hexagons. The green and grey regions in the diagram indicate the configurations for which the ground state is a low-spin state. The red and yellow regions show the configurations with a spin polarized ground state. The largest energy gap $\Delta E=-230\,\mu$eV occurs at $\mu _y=5.2$ nm and $\mu _x-\mu _y=1.6$ nm and is indicated with the most saturated red color.

The energy gap $\Delta E$ for various geometry of the quantum dot array is given in a phase diagram that displays the nonferromagnetic and Nagaoka ferromagnetic phases in Fig. 4. The diagram is calculated for a small magnetic field of 1 mT. For the case with $\mu _y\ge 7$ nm, the four dots are located far away from each other, rendering tunneling negligible, and hence the lowest spin $S=1/2$ and spin $S=3/2$ states are nearly degenerate, as seen on the right side of the figure. In the case where the dots are located much closer to each other in *y* direction (left side of the diagram), the ground state tends to be the spin-1/2 state as in Fig. 3c. This is a quantitative result for the strong tunnel coupling forming two double-dot subsystems. The region in between corresponds to the extended ferromagnetic system with a spin-polarized ground state. In this region, the tunneling between neighbouring dots is enough for the ’hole’ to move around the four dots. The largest gap is $\Delta E=-230\,\mu$eV and is located at $\mu _x=6.8$ nm, $\mu _y=5.2$ nm.

**Figure 5:**
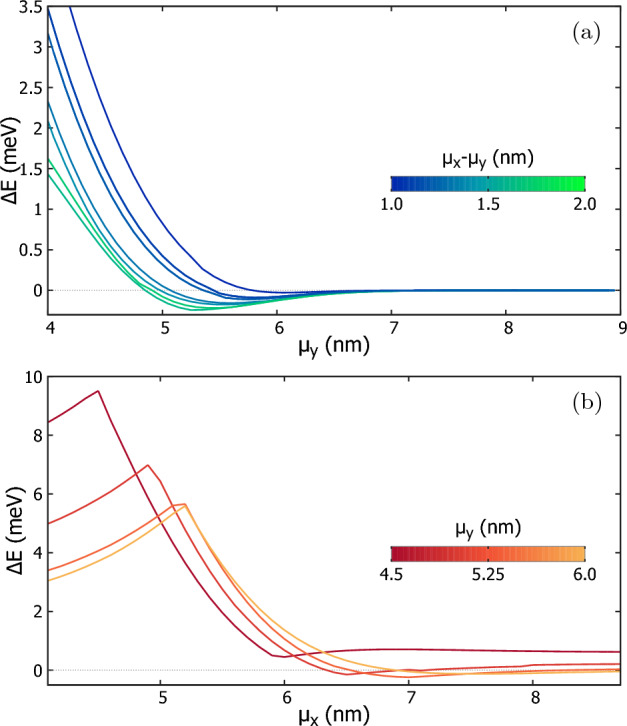
(**a**) The Nagaoka gap as a function of the parameters $\mu _y$ for different values of $(\mu _x-\mu _y)$. (**b**) as a function of parameter $\mu _x$ for various $\mu _y$’s. The lines shift towards the negative sides as the system parameters approach the largest Nagaoka gap point, after which the lines in both subfigures approach zero.

To understand in detail the effects of the interactions depending on the location of the dots, we study the cross sections of the phase diagram to see the energy gap as a function of the parameter $\mu _y$ for various $(\mu _x-\mu _y)$ in Fig. 5a. The top-most (bluest) line shows the change in the energy gap $\Delta E$ with $\mu _y$ for $\mu _x-\mu _y=1$ nm. It barely goes below the zero line, indicating a very weak Nagaoka ferromagnetic phase near $\mu _y=6$ nm. As the difference $\mu _x-\mu _y$ increases, the next four lines from the top slowly start to go much lower than the zero line, indicating that the system is in a stronger ferromagnetic phase and that it would take more energy to invert one of the spins and break the phase. The lowest line is the one that goes the lowest under the zero line, which is for the difference $\mu _x-\mu _y=1.6$ nm. The second line from the bottom (greenest) is for the difference of 1.9 nm and is now higher than the difference of 1.6 nm. At this point, the electron density begins to take the shape of two dumbbells with decreasing *x*-direction overlap.

Fig. 5b shows the energy gap as a function of $\mu _x$ for different *y* spacings. The plot has few interesting features that give insight on the system. First, every plot has a peak at low values of $\mu _x$ slightly below the fixed $\mu _y$ distance. The initial growth of the energy gap at the left side of the plot is due to an extension of the region accessible for electrons that has a larger influence on the spin-unpolarized state than on the spin-polarized state for which the electrons cannot occupy the same location anyway. When the system is separated to single-electron islands maxima the lines dip to a certain value before tending to a constant value as $\mu _x$ increases. This constant itself tends to zero for the lines as the parameter $\mu _y$ becomes large, indicating a vanishing tunnel coupling. In order from topmost (red) line in Fig. 5b, for $\mu _y=4.5$ nm the energy gap is always positive and for this *y* spacing, the system never attains the ferromagnetic ordering. For other parameters, a ferromagnetic ground state is found for a range of $\mu _x$.

**Figure 6:**
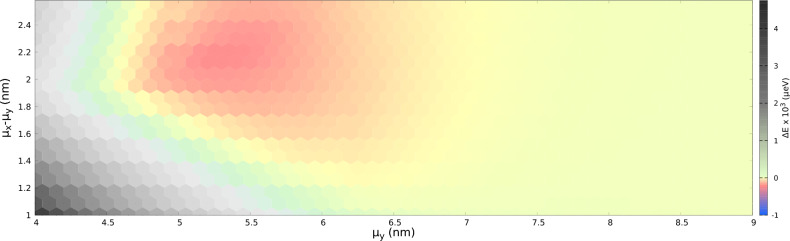
The energy gap, $\Delta E$ as a function of the parameters $\mu _y$ and $(\mu _x-\mu _y)$ with potential depth, $V_d=60$ meV. The energy gap is calculated for every point at the center of the hexagons. The largest energy gap $\Delta E=-211\,\mu$eV occurs at $\mu _y=5.35$ nm and $\mu _x-\mu _y=2.21$ nm. The diagram is similar as Fig. 4, but the lower potential depth has resulted in a major shift of the largest Nagaoka gap up on the $\mu _x-\mu _y$ scale and a minor shift on $\mu _y$ scale.

The maximal Nagaoka gap is expected to shift on the $\mu _x,\mu _y$ plane for a varied potential depth that affects the strength of the tunnel coupling. The phase diagram for the potential depth of $V_d=60$ meVis plotted in Fig. 6. The results are qualitatively similar to the case of $V_d=125$ meV presented above. The largest Nagaoka gap $\Delta E = -211\,\mu$eV occurs for the parameters $\mu _x=7.52$ nm and $\mu _y=5.35$ nm. Because of the shallower potential, the electrons are less localized within the separate dots. This results in a larger tunnel coupling and the Nagaoka phase is achieved for larger inter-dot distances compared to the case of $V_d=125$ meV. Note that the parameter $\mu _x$ changed from 6.8 nm to 7.5 nm, while the parameter $\mu _y$ changed only about 0.15 nm. This can be attributed to the heavier electron effective mass in *y* direction and the tunneling energy changing more strongly with the interdot distance.

### Shift from the rectangular geometry

**Figure 7:**
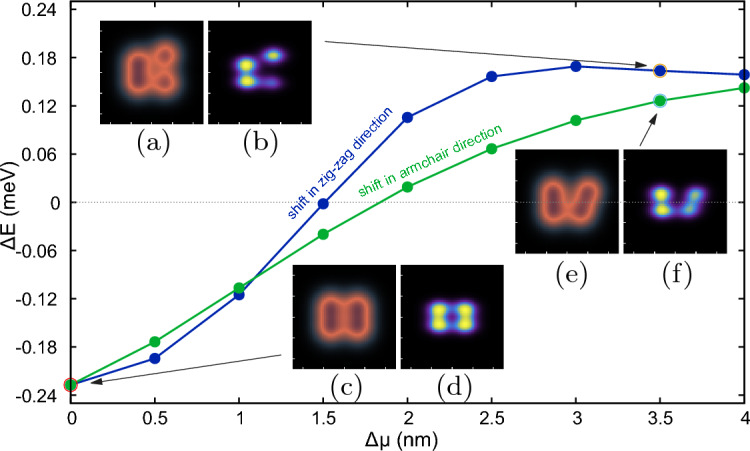
The Nagaoka gap as a function of shift $\Delta \mu$ for both in armchair direction (shown in blue) and zigzag direction (shown in green) from the starting point of $\mu _x=6.8$ nm, $\mu _y=5.2$ nm. The insets (**a**,**c**,**e**) show the external potential and insets (**b**,**d**,**f**) show the square root of ground state electron density. The insets (**a**,**b**) are for shift $\Delta \mu _x=3.5$ nm and (**e**,**f**) are for shift $\Delta \mu _y=3.5$ nm. The starting potential and square root density are shown in insets (**c**,**d**). The colors scale for insets is same as in Fig. (2). The difference in the curves can be explained using different mechanisms as discussed in Section “Shift from the rectangular geometry”.

In order to investigate the robustness of the spin-polarized ground state we deform the rectangular arrangement of dots so that electrons are trapped in a finite pseudo-1D chain. Starting from the initial state with the largest energy gap, i.e. $\mu _x=6.8$ nm, $\mu _y=5.2$ nm, we shift the location of the top-right dot in both *x* and *y* directions separately. The energy gap as a function of the shift $\Delta \mu$ is plotted in Fig. 7. The anisotropy in the effective mass makes the nature of Nagaoka transition different in armchair (*x*) and zigzag (*y*) direction. For a shift in armchair direction, the spin-1/2 states have much lower energy than the high-spin state and the electrons from a 1D pseudo chain structure as seen in inset (f) of Fig. 7. The electron occupancy of the lower right dot is low for the shift of $\Delta \mu _y=3.5$ nm in the zigzag direction. One of the electrons get fixed in the shifted dot, which is far away from the rest, which minimizes the Coulomb interaction energy. The two remaining electrons occupy the deeper left dumbbell instead of the shallower lower right dot [see inset (a) and (b) of Fig. 7]. No tunneling is possible between the rightmost dots and only a trace tunneling in the topmost dots. A similar effect is seen in the *x*-direction shift, but the geometry of the dots is such that the electrons form a chain with no tunneling between the two upper dots. Ref.^24^ showed that when the dot arrays are deformed to form a quantum dot chain, the spin polarization in the ground state is excluded by the Lieb-Mattis theorem^40^, which restricts the ground state solutions of such a 1D chain to low spin values. However, the anisotropy of the masses leads to an asymmetry in the tunneling of electrons between the dots. In the case of a shift in the zigzag direction, the system can be divided into two parts: the left double-dot subsystem and two single dots. As in the case of Fig. 3c, the double dot holds the singlet state of two electrons, and the third electron lowers the Coulomb repulsion by occupying the shifted dot. Tunnel coupling in the *y* direction decreases rapidly due to the higher effective mass, resulting in a transition occurring at a shift of about $\sim 1.5$ nm. In contrast, lifting the Nagaoka ground-state polarization requires a shift of approximately 1.85 nm in the *x*-direction.

### Potential detuning

**Figure 8:**
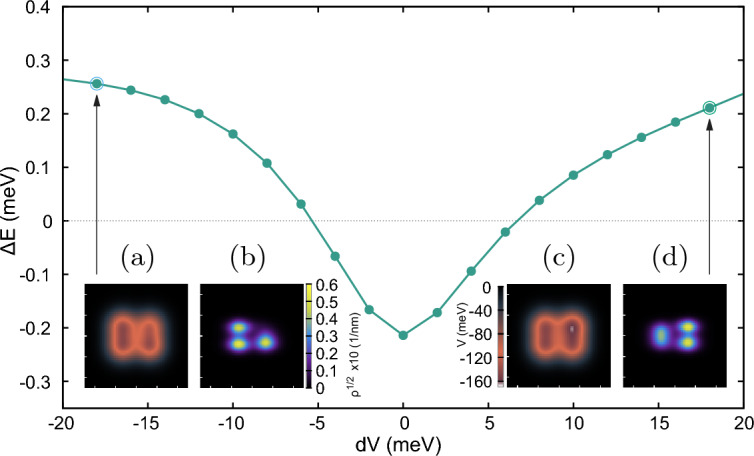
The Nagaoka gap as a function of an alteration in the potential of one of the dots, *dV* (meV). The insets (**a**, **b**) show the potential and square root electron density for value $dV=-18$ meV and insets (**c**,** d**) show the potential and the square root electron density for change in potential of $dV=18$ meV. The color scale for the potential and square root of density is shown. Unlike the case in Section “Shift from the rectangular geometry”, the anisotropy of the curve is inherent and is not unique to phosphorene.

In addition to moving the dots, it is possible to assess the tolerance of the ferromagnetic state to disorder by changing the potential depth of one of the dots. We vary the potential depth $V_d$ of just the top right dot by an amount *dV*. Fig. 8 shows the Nagaoka gap as a function of the change *dV* in the range $-20$ meV to 20 meV. The changes in the electron density as the potential changes are also shown in the insets of Fig. 8.

The most evident feature of the plot in Fig. 8 is the asymmetry in the energy gap variation for negative and positive potential changes. More precisely, for the positive change *dV* the Nagaoka ferromagnetic transitions to the low-spin state at $dV \approx 7.0$ meV, while the same transition occurs at a bit smaller change of $dV \approx -5.4$ meV for the negative change. The transition to the low-spin ground state for positive detuning is due to the localization of an electron in the detuned dot and the transition for negative detuning is due to the delocalization of an electron from the detuned dot which is excluded from the array by the energy mismatch leaving the three dots with an exact half-filling. When the top right dot is made deeper [insets (c) and (d)] the electron occupancy of this dot becomes larger than 1. When the dot is made shallower [insets (a) and (b)] the dot is emptied. In both cases the conditions for observation of the itinerant ferromagnetism are lifted, and the ground state acquires the low spin.

## Summary and conclusions

We have investigated the Nagaoka ferromagnetic state in the phosphorene quantum-dot plaquette using an effective mass Hamiltonian nearly half-filled system. We determined the geometry of the plaquette for the maximal stability of the spin-polarized ground state. For the chosen parameters of the isotropic single-dot Gaussian confinement the largest Nagaoka gap of $\Delta E=-230\,\mu$eV occurs for the parameters $\mu _x=6.8$ nm and $\mu _y=5.2$ nm. Shifting one of the dots in *x* or *y* direction breaks the ferromagnetic ordering of spins stopping the carrier hopping between now nonequivalent locations and forming a quasi-1D chain with low spin ground state on the ground of the Lieb-Mattis theorem. Our results indicate that the Nagaoka state in the phosphorene quantum dots exhibits strong resistance to disorder, which we tested in the form of detuning one of the dots. We showed that the mechanisms breaking the Nagaoka ordering are different for positive and negative detuning, and hence the transition occurs for uneven values of detuning.

## Data Availability

The data that support the findings of this study are available from the first author (T.T.) upon reasonable request.
